# Changes in the Shape and Volume of Subcortical Structures in Patients With End-Stage Renal Disease

**DOI:** 10.3389/fnhum.2021.778807

**Published:** 2021-12-16

**Authors:** Wen Gu, Ronghua He, Hang Su, Zhuanqin Ren, Lei Zhang, Huijie Yuan, Ming Zhang, Shaohui Ma

**Affiliations:** ^1^Department of Medical Imaging, The First Affiliated Hospital of Xi’an Jiaotong University, Xi’an, China; ^2^Department of Radiology, Baoji Center Hospital, Baoji, China; ^3^Department of Radiology, Baoji High-Tech Hospital, Baoji, China

**Keywords:** end-stage renal disease, magnetic resonance imaging, subcortical, shape analysis, volumetric analysis

## Abstract

**Introduction:** End-stage renal disease (ESRD) typically causes changes in brain structure, and patients with ESRD often experience cognitive and sleep disorders. We aimed to assess the changes in the subcortical structure of patients with ESRD and how they are associated with cognitive and sleep disorders.

**Methods:** We involved 36 adult patients for maintenance hemodialysis and 35 age- and gender-matched control individuals. All participants underwent neuropsychological examination and 3T magnetic resonance imaging (MRI) to acquire T1 anatomical images. The laboratory blood tests were performed in all patients with ESRD close to the time of the MR examination. We used volumetric and vertex-wise shape analysis approaches to investigate the volumes of 14 subcortical structural (e.g., bilateral accumbens, amygdala, hippocampus, caudate, globus pallidus, putamen, and thalamus) abnormalities in the two groups. Analyses of partial correlations and shape correlations were performed in order to identify the associations between subcortical structure, cognition, and sleep quality in patients with ESRD.

**Results:** The volumetric analysis showed that compared with the healthy control group, patients with ESRD had less bilateral thalamus (left: *p* < 0.001; right: *p* < 0.001), bilateral accumbens (left: *p* < 0.001; right: *p* = 0.001), and right amygdala (*p* = 0.002) volumes. In the vertex-wise shape analysis, patients with ESRD had abnormal regional surface atrophy in the bilateral thalamus, right accumbens, left putamen, and bilateral caudate. Moreover, the Montreal Cognitive Assessment (MoCA) score was associated with volume reduction in the bilateral thalamus (left: Spearman ρ = 0.427, *p* = 0.009; right: ρ = 0.319, *p* = 0.018), and the Pittsburgh Sleep Quality Index (PSQI) score was associated with volume reduction in the bilateral accumbens (left: ρ = −0.546, *p* = 0.001; right: ρ = −0.544, *p* = 0.001). In vertex-wise shape correlation analysis, there was a positive significant correlation between regional shape deformations on the bilateral thalamus and MoCA score in patients with ESRD.

**Conclusion:** Our study suggested that patients with ESRD have subcortical structural atrophy, which is related to impaired cognitive performance and sleep disturbances. These findings may help to further understand the underlying neural mechanisms of brain changes in patients with ESRD.

## Introduction

End-stage renal disease (ESRD), also known as uremia, is defined by a glomerular filtration rate of <15 ml/min/1.73 m^2^ (with the persistence of <10% renal function) or the development of chronic kidney disease (CKD) to stage 5 (Meyer and Hostetter, 2007; Jha et al., 2013). Patients with uremia require dialysis to maintain kidney function (Himmelfarb and Ikizler, 2010). Although hemodialysis and peritoneal dialysis can significantly prolong the survival of patients with ESRD, dialysis does not resolve complications experienced by patients with ESRD (Mettang and Kremer, 2015), which seriously affects the quality of life of patients (Zhang et al., 2012).

Patients with ESRD often have varying degrees of brain damage. According to the kidney–brain axis and cross-talk theory, both structural and functional changes are observed in the brain of patients with ESRD (Bugnicourt et al., 2013; Lu et al., 2015). The underlying pathophysiology of comorbid neurological diseases in patients with ESRD is related to the shared anatomical and vascular regulation systems and the bidirectional pathways of humoral and non-humoral systems that influence the kidneys and the brain. Therefore, different degrees of subcortical structural volume abnormalities are often observed in patients with ESRD. Although studies investigating these alterations have been conducted, results are inconsistent (Zhang et al., 2013; Chang et al., 2017; Jin et al., 2020). Recently, subcortical structural atrophy was reported in patients with ESRD who had been treated using hemodialysis (Prohovnik et al., 2007; Chiu et al., 2019). However, the effect of subcortical structural abnormalities on cognition and sleep in patients with ESRD remains unknown. Cognitive control deficits and sleep disturbance are common symptoms experienced by patients with ESRD (Scherer et al., 2017; Drew et al., 2019). Data have shown that the incidences of insomnia in patients undergoing maintenance hemodialysis and peritoneal dialysis are 79 and 73%, respectively, whereas the incidence of insomnia in the general population is only 12% (Hui et al., 2000, 2002). Moreover, approximately 60% of patients with ESRD have cognitive dysfunction (Kalirao et al., 2011). Various subcortical structural abnormalities have been reported in patients with cognitive impairment (Chang et al., 2017; Chiu et al., 2019) and insomnia (Alperin et al., 2019; Li et al., 2019). In fact, the kidney–brain axis and cross-talk theory suggested that subcortical structural volume abnormalities contribute to impaired sleep and cognition in patients with ESRD (Lu et al., 2015; Miranda et al., 2017).

Numerous methods can be used to study the volume and morphology of subcortical structures, which include, but are not limited to, vertex-wise analysis and voxel-based morphometry (VBM). Among these methods, vertex-wise shape analysis is a fully automated segmentation method that can provide the location and pattern of shape changes in subcortical structures. Furthermore, this method has advantages in detecting morphological changes of subcortical structures and is not dependent on tissue classification methods or arbitrary smoothing. Therefore, it can precisely locate the regional atrophy of the subcortical structure and detect the structural changes (Patenaude et al., 2011). At present, shape analysis is a precise approach for exploring atrophy-related lesions. It is used widely in the investigations of neurological diseases, such as Parkinson’s disease (PD) (Nemmi et al., 2015), temporal lobe epilepsy (TLE) (Weng et al., 2020), chronic insomnia disorder (Gong et al., 2019), and Alzheimer’s disease (AD) (de Jong et al., 2008). To date, only few studies have applied these methods to investigate subcortical structures in patients with ESRD.

In this study, we used vertex-wise shape analysis to compare the shape changes of subcortical structures between patients with ESRD and matched healthy controls (HCs). In addition, we also measured the volumes of the 14 subfields of the subcortical structure to compare the two groups. Analyses of partial correlations and shape correlations were then performed in order to detect associations between subcortical structural volume and neuropsychology in patients with ESRD.

## Materials and Methods

### Participants

The study was approved by the Ethics Committee of the First Affiliated Hospital of Xi’an Jiaotong University. After receiving a full explanation of the study, all participants provided an informed consent before participation.

In this study, we involved 36 patients with ESRD (26 males, 10 females; age range 25–65 years; mean age 43.64 years), and 35 healthy age- and gender-matched right-handed HCs (20 males, 15 females; age range 20–64 years; mean age 43.60 years), who underwent neuropsychological and magnetic resonance imaging (MRI) examinations, and laboratory blood tests were performed before single hemodialysis within 24 h. The underlying cause of all ESRD in patients was glomerulonephritis. The diagnosis of ESRD was based on serum creatinine levels (Stevens and Levin, 2013).

Inclusion criteria for the ESRD group were as follows: (a) age >18 years; (b) confirmed clinical diagnosis of ESRD (estimated glomerular filtration rate <15 ml/min/1.73 m^2^); and (c) on maintenance hemodialysis for more than 3 months.

Exclusion criteria for the ESRD group were as follows: (a) kidney transplantation; (b) history of drug or alcohol abuse; (c) severe neurological disease, psychiatric disorder (other than cognitive impairment), or organic brain disease (e.g., brain tumor and stroke); (d) severe complications, such as advanced liver, infections, and heart failure; (e) diabetic nephropathy or primary hypertensive nephropathy; (f) history of neurodegenerative disease (e.g., PD or AD); and (g) MRI examination contraindications (e.g., cardiac pacemaker implants or claustrophobia).

### Neuropsychological Assessments

The Pittsburgh Sleep Quality Index (PSQI) was used to evaluate sleep quality in the ESRD group (Buysse et al., 1989; Backhaus et al., 2002). The Montreal Cognitive Assessment (MoCA) was employed to evaluate cognitive function (Nasreddine et al., 2005). All neuropsychological assessments were carried out 1 h before MRI scanning was performed.

### Laboratory Blood Tests

Laboratory blood tests of patients with ESRD have included serum creatinine, serum blood urea nitrogen, cystatin-C, hemoglobin, and hematocrit. All laboratory variables were obtained close to the time of MR imaging. All blood samples were collected in the same laboratory using the same method. Blood samples were not collected from the HC group.

### Image Acquisition

All MRI structural images were acquired using a 3.0-Tesla MRI scanner (GE Discovery MR750w Healthcare, Milwaukee, WI, United States) equipped with an eight-channel phased-array coil. High-resolution T1-weighted scans of the whole brain were acquired for each participant using a 3D BRAVO (brain volume) pulse sequence with the following parameters: repetition time 8.2 ms, echo time 3.1 ms, 12° flip angle, 256 × 256 matrix resolution (voxel size: 1 mm × 1 mm × 1 mm), and 140 contiguous slices. Scans were used to conduct shape analysis of the subcortical structures. The conventional T2-weighted imaging and fluid-attenuated inversion recovery sequences were obtained in our study design to exclude severe neurological disease, psychiatric disorder, or organic brain diseases.

### Image Preprocessing

Segmentation of all subcortical structure and volume calculations were performed using FreeSurfer Version 6.0 (Fischl, 2012). Preprocessing of the imaging data mainly included the following steps: motion correction and intensity normalization (Reuter et al., 2010); labeling the volumes of each segmentation (Fischl et al., 2002); and removal of non-brain tissue using skull-stripping (Ségonne et al., 2004). We then applied the Bayesian algorithm to measure subcortical structural volume and Talairach registration (Fischl et al., 2004). Finally, manual quality checks were performed on the data by trained researchers.

Furthermore, we also used estimated total intracranial volume (eTIV) of all the subjects as a covariate in the subsequent analysis, which was calculated using SIENAX (Smith et al., 2004), a part of the FMRIB Software Library (FSL version 6.0.3^1^; Oxford University, Oxford, United Kingdom) (Jenkinson et al., 2012).

FMRIB’s Integrated Registration and Segmentation Tool (FIRST) (Patenaude et al., 2011) is a model-based segmentation and registration module contained in FSL (Jenkinson et al., 2012). The shape and appearance of the subcortical structures used in this method were established from 336 manually labeled brain images provided by the Center for Morphometric Analysis, Massachusetts General Hospital, Boston. FIRST uses a deformable mesh model to create a surface mesh that consists of a set of triangles for each subcortical structure, and the vertices of adjacent triangles are called vertices. Therefore, this approach, which is based on a Bayesian framework model, was used to process the data. Briefly, images were registered to Montreal Neurological Institute 152 space; all subcortical structures were constructed subcortical structures were automatically segmented; and the quality of segmentation was manually checked for every participant. Then, the outcome file output from FIRST was used for the vertex analysis.

### Statistical Analysis

Statistical analysis was performed using IBM SPSS Statistics (version 26.0 IBM Corp., Armonk, NY, United States). A two-sample *t*-test was used to compare various demographic data between the ESRD and HC groups, and a Mann–Whitney *U* test was used for non-normally distributed data. The Chi-squared test was used to analyze gender differences between the two groups. In addition, we used the Kolmogorov–Smirnov test to evaluate normality and Levene’s test to determine the equality of variances.

An analysis of covariance (ANCOVA) was used to estimate group differences in subcortical structural volume, with age, gender, and eTIV as covariates. Partial eta squared was a proportion of variance accounted for by effect, and we also calculated their values (Chen et al., 2017). Pearson correlations were performed to examine the relationships between the results of neuropsychological test and laboratory blood test and subcortical structure volume in the ESRD group, and the Spearman correlations were used for non-normally distributed data. Partial Pearson correlations were performed to examine the relationships between the results of neuropsychological test and laboratory blood test and subcortical structure volume in the ESRD group after controlling for the effects of age, gender, and eTIV, and partial Spearman correlation coefficients (ρ) were used for non-normally distributed data. Bonferroni correction was performed for the results of ANCOVA [*p* < 0.004 (0.05/14) was considered statistically significant] and correlation analysis [*p* < 0.025 (0.05/2) was considered statistically significant].

A permutation-based non-parametric analysis of subcortical shape data and general linear models comprise age and gender as covariates (Winkler et al., 2014). Results were corrected for multiple comparisons using threshold-free cluster enhancement and FWE methods (*p* < 0.05) (Smith and Nichols, 2009).

## Results

### Demographic, Clinical, and Neuropsychological Data

The MoCA and PSQI scores were non-normally distributed data. Demographic information and clinical characteristics of participants are summarized in Table 1. There were no significant differences in age (*p* = 0.989), gender (*p* = 0.184), or education (*p* = 0.115) between patients with ESRD and HCs. Patients with ESRD had significantly lower MoCA scores (*p* < 0.001) and had higher PSQI scores (*p* < 0.001) compared with HCs.

**TABLE 1:** Demographic and clinical characteristics of participants.

Variables	ESRD (*n* = 36)	HC (*n* = 35)	*p*-Value	Comparison
Age (years)	43.64 (11.37)	43.60 (12.35)	0.989^a^	NS
Gender (male/female)	26/10	20/16	0.184^b^	NS
Education (years)	10.78 (2.55)	11.63 (1.89)	0.115^a^	NS
Dialysis duration (months)	27.89 (26.11)			
Laboratory blood tests				
Blood urea nitrogen				
(mmol/L)	22.81 (8.80)			
Creatinine (μmol/L)	800.33 (250.99)			
Cystatin-C (mg/L)	5.00 (1.58)			
Hemoglobin (g/L)	94.53 (19.44)			
Hematocrit	27.95 (9.00)^d^			
Psychometric				
MoCA (scores)	25.00 (6.00)^d^	28.00 (1.00)^d^	<0.001^c^	HC > ESRD
PSQI (scores)	7.50 (4.00)^d^	4.00 (2.00)^d^	<0.001^c^	HC < ESRD

*PSQI, Pittsburgh Sleep Quality Index; MoCA, Montreal Cognitive Assessment; NS, not significant; ESRD, end-stage renal disease; HC, healthy controls; SD, standard deviation; IQR, interquartile range.*

*Data are shown as means (SDs).*

*^a^Independent-samples t-test was used for data analysis.*

*^b^χ^2^ test was used for data analysis.*

*^c^Mann–Whitney U test was used for data analysis.*

*^d^Medians (IQRs).*

### Subcortical Volume Analyses

**FIGURE 1:**
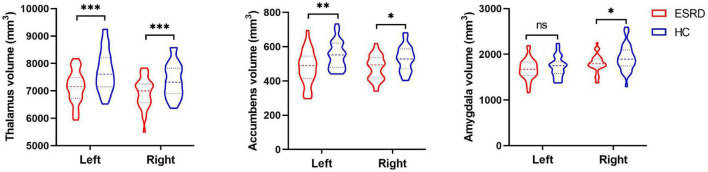
The violin plot depicts significantly different subcortical structural volumes between patients with end-stage renal disease (ESRD) and healthy controls (HCs), which included the bilateral thalamus, bilateral accumbens, and right amygdala, after adjustment for age, gender, and estimated total intracranial volume. Red color represents patients with ESRD, and blue color represents HCs. Inner violin plot shows the quartile and the median. *Significance after Bonferroni correction. *p < 0.05/14, ^**^p < 0.01/14, ^***^p < 0.001/14; ns, not significant.

After Bonferroni correction, subcortical volumes are significantly different between the ESRD and HC groups in the bilateral thalamus (left: *F* = 31.479, *p* < 0.001; right: *F* = 22.820, *p* < 0.001), bilateral accumbens (left: *F* = 15.321, *p* < 0.001; right: *F* = 13.148, *p* = 0.001), and right amygdala (*F* = 9.924, *p* = 0.002), as shown in Table 2. Compared with the HC group, the ESRD group had greater volume atrophy in the bilateral thalamus, bilateral accumbens, and right amygdala (Figure 1).

**TABLE 2:** Results of subcortical volumes group comparisons.

	ESRD Mean volumes (SD) (mm^3^)	HC Mean volumes (SD) (mm^3^)	*F*	Partial eta squared	*p*-Value
**Left thalamus**	**7135.78 (595.41)**	**7719.50 (732.23)**	**31.479**	**0.323**	**<0.001**
Left caudate	3652.33 (420.38)	3596.26 (336.63)	0.074	0.001	0.787
Left putamen	4960.81 (428.24)	5117.30 (420.89)	6.002	0.083	0.017
Left globus pallidus	1825.13 (188.20)	1857.63 (130.90)	4.019	0.049	0.057
Left hippocampus	3936.08 (337.32)	4106.19 (353.27)	8.554	0.115	0.005
Left amygdala	1676.83 (219.50)	1731.37 (222.25)	2.575	0.038	0.113
**Left accumbens**	**478.63 (98.61)**	**549.65 (79.19)**	**15.321**	**0.188**	**<0.001**
**Right thalamus**	**6940.78 (534.16)**	**7180.01 (618.19)**	**22.820**	**0.257**	**<0.001**
Right caudate	3788.63 (533.11)	3678.91 (349.31)	0.388	0.006	0.536
Right putamen	4993.29 (472.63)	5113.54 (476.52)	3.059	0.044	0.085
Right globus pallidus	1816.54 (230.91)	1858.42 (154.08)	2.870	0.042	0.095
Right hippocampus	4186.49 (300.52)	4327.37 (383.52)	4.151	0.046	0.059
**Right amygdala**	**1797.43 (195.44)**	**1926.83 (283.57)**	**9.924**	**0.131**	**0.002**
**Right accumbens**	**477.57 (70.72)**	**530.40 (72.61)**	**13.148**	**0.166**	**0.001**

*eTIV, estimated total intracranial volume.*

*Bold figures represent significant volumetric reductions after adjustment for age, gender, and eTIV. Threshold for significance α = 0.004 (0.05/14) after Bonferroni correction.*

### Subcortical Shape Analyses

**FIGURE 2:**
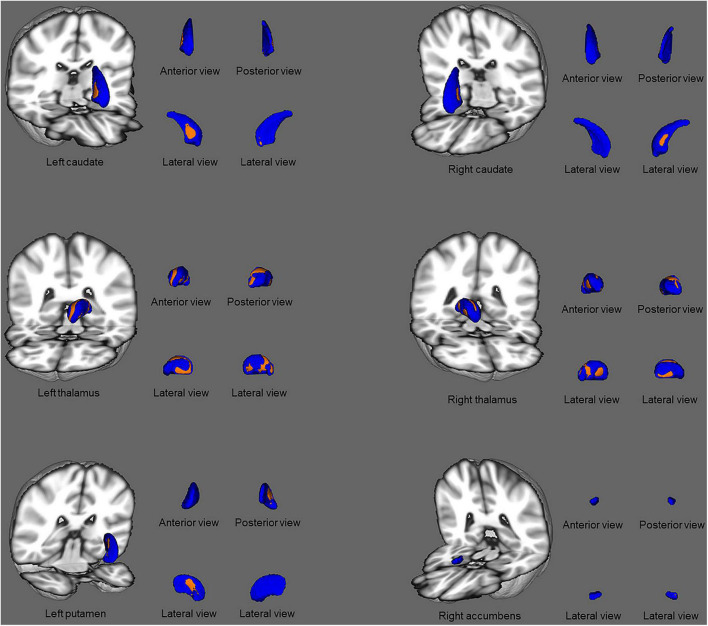
Subcortical structural shape differences between the ESRD group and healthy controls using vertex-wise surface analyses. Subcortical surface alterations were observed in the bilateral thalamus, right accumbens, left putamen, and bilateral caudate, after adjustment for age and gender. The blue models indicate the three-dimensional subcortical structure mesh, and the orange layers represent the significant reductions. Results were corrected for multiple comparisons using threshold-free cluster enhancement and family-wise error methods (*p* < 0.05).

Comparisons of the vertex-wise shape of subcortical structures between patients with ESRD and HCs are shown in Figure 2. Significantly different subcortical structures are shown in a three-dimensional model: the blue models represent the subcortical structures and the orange layers represent regions with significant differences. Automated subcortical structure vertex-wise analysis revealed significant subcortical volumetric reductions in patients with ESRD in the bilateral thalamus, right accumbens, left putamen, and bilateral caudate. However, in contrast to the results of the subcortical volume analysis, there were no significant differences between groups in the left accumbens and the right amygdala.

### Correlation Analyses

**FIGURE 3:**
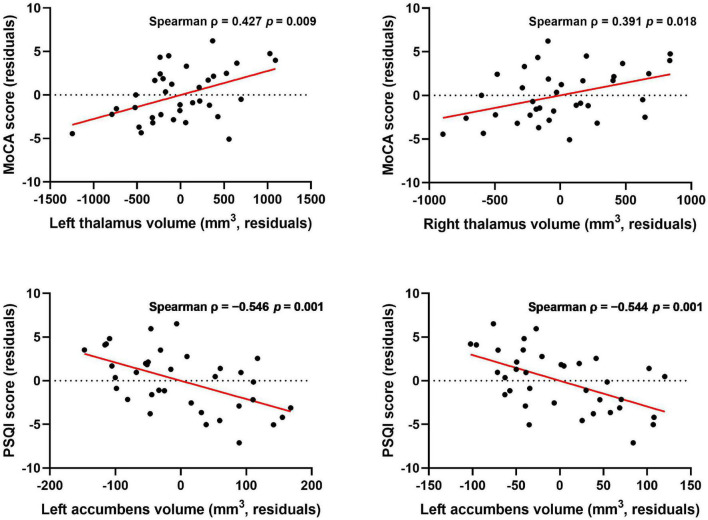
Association between subcortical structure volume and MoCA and PSQI scores after controlling for age, gender, and eTIV. Thalamus volume was correlated with MoCA score, and accumbens volume was correlated with PSQI score after controlling for age, gender, and eTIV [*p* < 0.025 (0.05/2) was considered statistically significant after Bonferroni correction].

In the ESRD group, there was a significant correlation between the bilateral thalamus volume and MoCA score (left: Spearman ρ = 0.427, *p* = 0.009; right: ρ = 0.391, *p* = 0.018; Figure 3) by correlation analyses after adjustment for age, gender, and eTIV. Similarly, there was also a significant negative correlation between the bilateral accumbens volume and PSQI score after adjustment for age, gender, and eTIV (left: ρ = −0.546, *p* = 0.001; right: ρ = −0.544, *p* = 0.001; Figure 3). In addition, there was a significant correlation between the left thalamus volume and serum creatinine after adjustment for age, gender, and eTIV (*r* = −0.518, *p* = 0.002). Moreover, there were no significant correlations between right amygdala volume and neuropsychological scores (i.e., PSQI or MoCA scores) after adjustment for age, gender, and eTIV. There was no correlation between the dialysis duration and the bilateral thalamus or bilateral accumbens volume after adjustment for age, gender, and eTIV.

**FIGURE 4:**
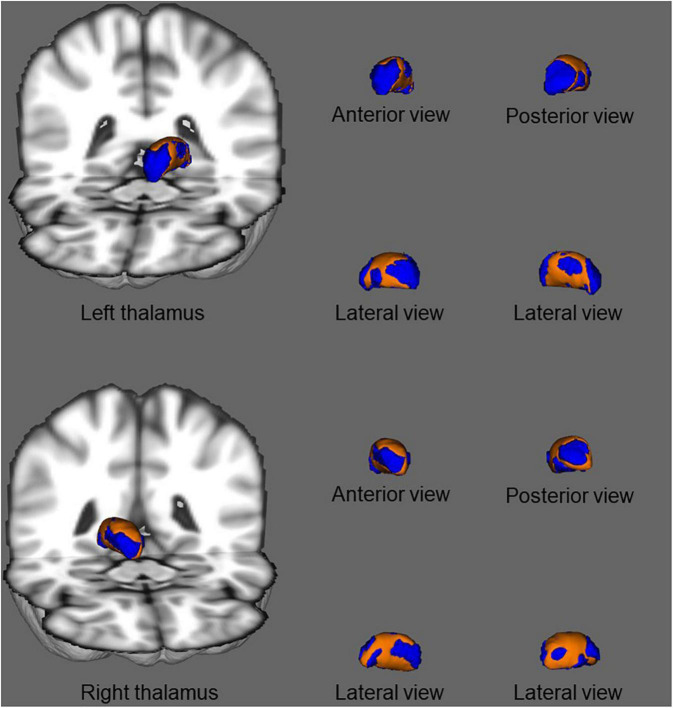
In shape analysis correlations, association between regional shape deformation on the ventral and dorsal side of the bilateral thalamus and MoCA score in patients with ESRD after adjustment for age and gender. The regions in orange color indicate the area associated with the MoCA score. Results were corrected for multiple comparisons using threshold-free cluster enhancement and family-wise error methods (*p* < 0.05).

In shape analysis correlations, there was a significant positive correlation between regional shape deformation on the ventral and dorsal sides of the bilateral thalamus and MoCA score in patients with ESRD after adjustment for age and gender (Figure 4). However, there were no correlations of PSQI score with regional shape deformation on the bilateral accumbens in patients with ESRD after adjustment for age and gender.

## Discussion

In this study, we investigated the relationship between changes in subcortical structural volume and cognitive and sleep symptoms in patients with ESRD using automated volumetric and vertex-wise shape analyses. We found atrophy of various subcortical structures in patients with ESRD, particularly the thalamus and accumbens. Moreover, in patients, cognition was associated with thalamic volume, and sleep quality had a trend relationship with accumbens volume. In addition, our results highlighted the impact that the changes in subcortical structures have on cognition and sleep of patients. Furthermore, shape analysis and volumetric analysis play a role in cross-validation, which can be used to study the changes of subcortical structures in patients with ESRD.

Abnormalities of subcortical structures have been reported previously in patients with ESRD, irrespective of dialysis status. Our study showed that patients with ESRD undergoing dialysis have atrophy of the bilateral thalamus, bilateral accumbens, and right amygdala. A recent study found the reduced volume of all subcortical structures in patients undergoing dialysis, including the thalamus, accumbens, and amygdala (Chiu et al., 2019). The study used the same processing software (FreeSurfer) to analyze the volume of the subcortical structure. Similarly, a previous study reported that patients with ESRD had reduced volume in the bilateral amygdala (Li A. et al., 2018). In addition, previous studies reported hippocampal volume atrophy in patients with ESRD (Chang et al., 2017). We also detected atrophy of the left hippocampus, albeit without Bonferroni correction. However, in contrast, it is different from our research results, and a recent study found significantly higher bilateral thalamus volumes in hemodialysis patients compared with HCs, although their results on the caudate and amygdala volumes were similar to our observations (Jin et al., 2020). Previous researchers found that compared with HCs, patients with minimal nephro-encephalopathy ESRD had lower brain volumes of the right amygdala and hippocampus, and those with non-nephro-encephalopathy ESRD had higher brain volumes of the right caudate and right thalamus (Zhang et al., 2013). However, the previous neuroimaging study included 19 hemodialysis patients and 14 HCs bilateral caudate volumes lower in patients (Prohovnik et al., 2007). These different findings are likely related to differences in study patients and methods used. For example, some researchers used VBM to analyze subcortical volumes instead of the volumetric analysis methods used in our study (Prohovnik et al., 2007; Zhang et al., 2013; Jin et al., 2020). Moreover, our study subjects differed from those of previous studies; the previous study selected patients with ESRD with non-nephro-encephalopathy, whereas the patients in our study had general cognitive and sleep disorders (Zhang et al., 2013). Furthermore, the use of stringent statistical correction may also contribute to inconsistent findings.

Vertex-wise analysis was used for the first time to study subcortical structural abnormalities in patients with ESRD undergoing maintenance hemodialysis, and we demonstrated its feasibility as an analysis method to detect atrophy. Using vertex-wise analysis, we found subcortical atrophy not only in the bilateral thalamus and accumbens and right amygdala but also in the bilateral caudate and left putamen. However, using volume analysis, we found that the volume of the bilateral thalamus, bilateral nucleus accumbens, and right amygdala decreased in patients with ESRD compared with the HC group. Similarly, inconsistent results between the two analysis methods were reported in a study exploring subcortical structures in patients with chronic insomnia (Gong et al., 2019) and alcohol dependence (Shim et al., 2019). Although the results of the vertex-wise shape analysis and volumetric analysis are different, they complement each other and play a role in cross-validation. In other words, these two methods are used to observe the changes of subcortical structures in patients with ESRD from different perspectives.

By combining vertex-wise and automated volumetric analysis results, we can study both volumetric and morphological abnormalities of the thalamus and accumbens in patients with ESRD. The vertex-wise analysis results showed that the atrophy shape of the thalamus roughly reflected the ventral anterior, mediodorsal, ventral posterior, and ventral lateral nuclei. Different areas of thalamic infarction can cause different cognitive impairments, such as a decline in memory and learning ability, lowered levels of consciousness, and decreased orientation, depending on the blood supply arteries (Chen et al., 2019). The anatomical and physiological bases of the kidney–brain axis and kidney–brain cross-talk hypothesis are related to the similarity in the structure and function of the blood supply arteries between the kidneys and the brain (Bugnicourt et al., 2013; Lu et al., 2015). Furthermore, a recent review reported that uremic neurotoxins that interact with the brain vasculature, neural progenitor cells, monoaminergic neurons, and the glymphatic system cause brain dysfunction in patients with CKD due to vascular insults and neurodegeneration (Viggiano et al., 2020). This was confirmed by a recent kidney transplant study, which demonstrated that the normalization of cerebral blood flow and neurochemicals in the bodies of patients with CKD after transplantation was related to improvements in cognition (Lepping et al., 2020). In fact, we observed that the MoCA score of patients with ESRD was positively correlated with bilateral thalamus volume, supporting the kidney–brain axis hypothesis. In addition, patients with ESRD have anemia that manifests as a decrease in hemoglobin and hematocrit (Kuwabara et al., 2002). Although we did not find a correlation between hemoglobin and hematocrit and thalamus and accumbens volumes in patients with ESRD, we observed a negative correlation between serum creatinine and volume of the left thalamus. This may carry some significance in terms of the relationship between the kidneys and the brain. Some scholars have also observed a similar relationship between serum creatinine and brain volume. They found that serum creatinine was negatively correlated with the volumes of right frontal lobe, left uncus, and left temporal pole (Zhang et al., 2013). It is suggested that the accumulation of major uremic toxins is one of the main risk factors for the change of volume of subcortical structures and the results are also consistent with the neurodegenerative disease in the kidney–brain axis hypothesis (Bugnicourt et al., 2013). Serum creatinine reflects the degree of renal impairment in patients with ESRD (Levey et al., 1999). The above results indicate that the severity of renal damage may be related to the changes in the volume of brain structures.

Similar findings were also revealed in the accumbens, and the volumetric analysis showed bilateral atrophy. Furthermore, slight atrophy was found in the right medial accumbens using vertex-wise analysis. The accumbens is a major component of the ventral striatum and is an area of continuity between the head of the caudate nucleus and the putamen; this region is primarily supplied by the recurrent artery of Heubner (Feekes and Cassell, 2006). The accumbens plays an important role in sleep and wakefulness and comprises two parts, namely, the shell and the core. Most neurons in the accumbens are gamma-aminobutyric acidergic neurons, which project fibers to the ventral pallidum, parabrachial nucleus, lateral hypothalamus, and ventral tegmental area, and the activation of these nuclei causes cortical awakening (Lazarus et al., 2012). Some researchers found that damage to both the core and shell of the accumbens causes a significant increase in wakefulness and a significant reduction in the sleep homeostatic response (Qiu et al., 2012). Similar to the thalamus volume results, we found that the severity of sleep disorders in patients with ESRD was related to the volume of the bilateral accumbens. In summary, based on the vascular insult and neurodegeneration hypothesis, direct neuronal toxicity, endothelial dysfunction, and vascular injury may cause subcortical atrophy, which would cause subsequent relative symptoms in patients with ESRD.

In addition, the relationship of morphometric alterations between thalamus or accumbens and the other network regions in patients with ESRD may also have value for exploration. Some studies have been reported the relationship between them in patients with TLE by the network of structural covariance (Zhang et al., 2017; Xu et al., 2020). This method can reveal the effect of changes in different subcortical gray matter (GM) nuclei on cortical GM in patients with ESRD.

Dialysis as a treatment measure does affect the state of a patient and will influence brain volumes, since this therapy will eliminate water from the subjects. Previous studies on single dialysis session in patients with ESRD focused on changes in the brain activity of a patient, and functional MRI (fMRI) was used in these studies (Li P. et al., 2018; Peng et al., 2021). Previous studies on the brain structure of patients with ESRD also did not involve the impact of a single dialysis (Li A. et al., 2018; Chiu et al., 2019; Jin et al., 2020). The possible reason for the different study methods mentioned earlier is that it is generally considered that changes in brain structure are a long-term process, and brain activity can change in a short time. Moreover, we found that there was no correlation between the dialysis duration and the bilateral thalamus volume or bilateral accumbens volume after adjustment for age, gender, and eTIV. This result is inconsistent with a previous study, which shows that the GM volume was strongly related to the duration of hemodialysis treatment on patients with ESRD (Prohovnik et al., 2007). The above results may be caused by the difference between the method (VBM vs. volumetric analysis) and the selected subject (global GM vs. thalamus). Therefore, the effect of a single dialysis session on changes in the brain structure of patients with ESRD may require further study in future.

The strengths of this study are as follows. First, vertex-wise analysis was used to study subcortical structures in patients with ESRD. Although this method has been applied to the study of subcortical structures in patients with various neurological diseases (de Jong et al., 2008; Nemmi et al., 2015; Gong et al., 2019), it has rarely been used in patients with ESRD. Second, the subcortical structures of ESRD patients were explored more objectively and clearly using vertex-wise shape analysis and the volumetric analysis than previous reports (Chang et al., 2017; Chiu et al., 2019; Jin et al., 2020). Third, we investigated correlations between subcortical structural changes and related symptoms, such as cognitive and sleep disorders.

However, the limitations of this study should also be mentioned. First, there were no non-dialysis-dependent ESRD patients and ESRD patients after a single dialysis as controls; therefore, it was not possible to determine the effect of dialysis on the subcortical structures of patients with ESRD. Thus, the patient group should be included in future studies. Second, the number of patients with ESRD was small, and a multicenter collaborative study is warranted. Third, we did not follow up patients; long-term longitudinal studies will help to understand the subcortical structural changes in patients with ESRD over time.

## Conclusion

We confirmed that patients with ESRD have atrophy of subcortical structures, especially the thalamus and accumbens. In addition, atrophy of the thalamus was associated with cognitive dysfunction, and accumbens atrophy was related to sleep disorders in patients with ESRD. These findings contribute to a deeper understanding of the mechanisms underlying the neurological symptoms of patients with ESRD.

## Data Availability Statement

The original contributions presented in the study are included in the article/supplementary material, further inquiries can be directed to the corresponding authors.

## Ethics Statement

The studies involving human participants were reviewed and approved by the Medical Ethics Review Board of the First Affiliated Hospital of the Medical College in Xi’an Jiaotong University. The patients/participants provided their written informed consent to participate in this study.

## Author Contributions

WG, SM, and MZ contributed to the research idea and study design. WG, HS, and RH performed the data acquisition and analysis. WG and RH performed manual quality checks on the data. WG and HY wrote this manuscript. LZ and ZR interpreted the data and managed the selection. All authors read and approved the final manuscript.

## Conflict of Interest

The authors declare that the research was conducted in the absence of any commercial or financial relationships that could be construed as a potential conflict of interest.

## Publisher’s Note

All claims expressed in this article are solely those of the authors and do not necessarily represent those of their affiliated organizations, or those of the publisher, the editors and the reviewers. Any product that may be evaluated in this article, or claim that may be made by its manufacturer, is not guaranteed or endorsed by the publisher.

## Footnotes

1
https://fsl.fmrib.ox.ac.uk/
